# Negative Pressure Wound Therapy (NPWT) after Hybrid Reconstruction of Occipital Pressure Sore Using Local Flap and Skin Graft

**DOI:** 10.3390/medicina59071342

**Published:** 2023-07-21

**Authors:** Seungchul Baek, Jun Ho Park

**Affiliations:** Department of Plastic and Reconstructive Surgery, SMG-SNU Boramae Medical Center, Seoul National University College of Medicine, Seoul 07061, Republic of Korea

**Keywords:** pressure sores, occipital scalp, negative pressure wound therapy, local flap, split-thickness skin graft

## Abstract

*Background and objectives:* Pressure sores are a common medical burden among patients, particularly those who are bedridden or frail. Surgical management of occipital pressure sores poses unique challenges due to limited elasticity and the spherical shape of the scalp. This study aims to evaluate the efficacy and safety of a novel reconstruction method utilizing a local transpositional flap and split-thickness skin graft with negative pressure wound therapy (NPWT) for occipital pressure sore treatment. *Material and methods:* A retrospective analysis was performed on patients with occipital pressure sores who underwent hybrid reconstructions using a local flap and split-thickness skin graft in conjunction with NPWT. Surgical outcomes, including flap survival rate, graft take percentage, and complications, were assessed. A comparative analysis was performed between the NPWT group and the conventional dressing group. *Results:* The NPWT group (*n* = 24) demonstrated a significantly higher mean graft take percentage at postoperative day 14 compared with the conventional dressing group (n = 22) (98.2% vs. 81.2%, *p* < 0.05). No significant difference in flap survival rate was observed between the two groups. *Conclusions:* As the aging population continues to grow, occipital pressure sores have gained significant attention as a crucial medical condition. The innovative surgical method incorporating NPWT offers an efficient and safe treatment option for patients with occipital pressure sores, potentially establishing itself as the future gold standard for managing this condition.

## 1. Introduction

Advances in medicine and healthcare have led to a remarkable increase in life expectancy worldwide. However, this longevity comes with its own set of challenges, including an increase in medical burdens. Among the various medical issues faced by older patients, pressure sores represent a common and significant burden. As individuals age and become frail, or when they suffer from specific conditions such as stroke, the vicious cycle of frailty can render them bedridden, making them highly susceptible to numerous health complications, including pressure sores. These sores develop as a result of sustained pressure on soft tissues, leading to hypoxic damage and eventual tissue necrosis [1].

To prevent pressure sores, it is important to either reduce the amount of strain or stress on tissues or to decrease the length of time that tissues are exposed to sustained strain or stress. Repositioning is one of the best prevention methods [1]. However, managing pressure sores proves to be a complex task due to the patient’s underlying frailty caused by factors such as malnutrition, sarcopenia, or the primary medical disease that necessitates their bedridden state [2]. While increased awareness, improved preventive measures, and earlier diagnosis contributed to a decline in the number of pressure injuries between 2012 and 2016, according to an international pressure ulcer prevalence survey, the prevalence of severe pressure injuries requiring surgical intervention has remained unchanged [3]. Although pressure sores can manifest in various regions of the body, they predominantly occur in areas with underlying bony prominences, such as the coccyx and trochanter. The posterior scalp, particularly in critical care settings with mechanically ventilated patients or individuals requiring head stabilization equipment such as neck collars or intracranial pressure-monitoring devices, also represents a common site for pressure sores [4].

The scalp has special feature unlike other tissue of the body. Skin has high density hair follicles and there is thick inelastic tissue, galea aponeurotica. The scalp holds significant importance as it not only safeguards the calvarium, but also carries cosmetic significance. With its abundant vascular supply, the scalp possesses an excellent healing potential, often necessitating surgical intervention only in severe cases of pressure sores. Surgical treatment options include debridement of necrotic tissue, followed by reconstruction using various techniques such as primary closure, skin grafting, local flap, regional flap, or free tissue transfer. However, scalp reconstruction presents unique challenges due to limited elasticity and specific characteristics that must be carefully considered. Factors such as defect size, depth, location, and patient condition play crucial roles in determining the appropriate surgical approach, with the reconstruction ladder serving as a valuable reference for decision making [5]. The optimal approach to scalp reconstruction entails closing the defect using hair-bearing tissue that closely matches the surrounding area.

In this study, we propose a novel and promising reconstruction method that utilizes a local transpositional flap and split-thickness skin graft, complemented by the application of negative pressure wound therapy (NPWT). Our research team successfully employed this technique to reconstruct occipital scalp defects, ensuring functional restoration and long-term stability. The efficacy and safety of this method were meticulously evaluated, showcasing its superiority over conventional approaches by offering better outcomes and relatively shorter surgical durations.

## 2. Materials and Methods

Our institutional review board (IRB No. 2020–10-150478) approved this retrospective review of patients with pressure sores on occiput using a local transposition flap with split thickness skin graft in conjunction with NPWT (negative pressure wound therapy). All patients included in this analysis provided written informed consent after three postoperative months at the outpatient clinic. The study was conducted in accordance with the Declaration of Helsinki and its later amendments. Informed consent was obtained from patients for all surgical procedures and wound management, and for the possible use of anonymized photographs. Eligible patients were those who underwent flap procedures (free flap and local flap) for occipital pressure sore treatment from March 2019 through December 2022. This was a single-center study, and a single attending plastic surgeon (J.H. Park) performed all of the procedures, which were carried out using standard methods.

- **Surgical Technique**

In cases where the patient presented with an occipital pressure sore accompanied by necrosis, necessitating debridement and reconstruction, a thorough assessment of the patient’s general condition was conducted in consultation with the anesthesia department. If the patient received an ASA score of 4 or 5, surgical intervention was contraindicated, and conservative treatment options were considered. However, if surgery was deemed appropriate, the patient’s hair was prepared by cutting it prior to the procedure. The surgery was performed under general anesthesia, and the patients were positioned in a prone orientation to optimize access to the affected region on the occipital scalp. Scalp preparation and draping were done aseptically.

At first, debridement was done. Necrotic tissue was removed until viable tissue appeared. The endpoint of debridement was the presence of fresh bleeding from the surface. After debridement using Metzenbaum scissors and a Bovie, a local transposition flap was meticulously elevated through dissection of the subgaleal plane. The local flap was a galeocutaneous flap, which is composed of skin, subcutaneous tissue, and galea. The periosteum remained in its original position. The flap design was performed using a random pattern, and the precise locations of key vessels, such as the occipital artery and superficial temporal artery, were not assessed prior to flap elevation. Repositioning of the flap was accomplished through either rotation or advancement, with the specific method and pivot angle varying from case to case. However, based on extensive surgical experience with the scalp, great care was taken to avoid excessive pivot angles and tension. The flap was meticulously inset to cover the exposed bone, securing it in place through suturing with adjacent viable scalp and periosteum at the original flap site. After the local flap transposition, the split thickness skin graft (thickness: 0.2 mm) was harvested from the posterolateral thigh. For a better aesthetic and functional outcome, Acellular dermal matrix (Alloderm, LifeCell Corp, Branchburg, NJ, USA) was used. Acellular dermal matrix and harvested split thickness skin were covered on the periosteum of the donor site of the galeocutaneous transposed flap. After all the procedures including flap inset and skin graft were performed, black NPWT sponge (V.A.C. Granufoam, KCI, now part of 3 M company, San Antonio, TX, USA) was then placed above wound margin of transposed local flap and grafted skin. The device (INFOV.A.C. Therapy Unit, KCI, now part of 3 M company, San Antonio, TX, USA) was set to −125 mmHg on continuous mode. NPWT apparatuses were removed after 7 postoperative days.

- **Postoperative Management**

Some patients were categorized into the NPWT management group. These patients underwent local flap coverage for defects, including a bony exposure area and split thickness skin graft on the periosteum, along with NPWT application. On the other side, other patients were categorized into the conventional dressing group. These patients underwent same surgical procedure as the NPWT group, except for the application of NPWT. The operative site was covered with antibiotic-ointment-coated Physiotulle (hydrocolloid-based, non-adherent wound contact layer, Coloplast, Ltd., Peterborough, UK) and the usual foam dressing material.

Immediately postoperatively, the sizes of the flap and graft surface were separately measured using the standard planimetry method. All of the initial surgical results were monitored closely after taking the dressing off by a single physician in the department of plastic and reconstructive surgery on the fourth postoperative day in the operation room. In the NPWT management group, NPWT was re-applied and was maintained until the 7th postoperative day. The dressing was changed to a conventional method (antibiotic ointment, Physiotulle, and foam) from the 8th postoperative day. The surgical outcomes were evaluated on the 14th postoperative day at the outpatient clinic. The surgical outcomes of interest were the rates of flap survival, graft take percentage, and complications (including infection, seroma, hematoma, and flap necrosis).

- **Statistical Analysis**

Statistical analyses were conducted using SPSS Statistics for Windows, version 26.0 (IBM Corp., Armonk, NY, USA). Categorical data were expressed as percentages. Continuous variables were expressed as the mean ± (standard deviation) for normally distributed variables or as the median for non-normally distributed variables. Continuous variables were compared using Mann–Whitney U test; categorical variables were compared using Fisher’s exact test. Statistical significance was defined by *p* values < 0.05.

## 3. Results

This study included 46 patients with a mean follow-up duration of 6.1 months for the NPWT group and 5.9 months for the conventional dressing group. Table 1 summarizes all of the other characteristics. Among the patients with occipital pressure sores, the NPWT group included 24 local flaps with skin grafts, and the conventional dressing group included 22. The mean flap surface area was 60.5 cm^2^ in the NPWT group and 57.2 cm^2^ in the conventional dressing group. The operation time was 85.7 min in the NPWT group and 80.4. min in the conventional dressing group. The difference was not statistically significant.

There was no significant intergroup difference in flap survival rate (100% in both groups). However, compared with the conventional dressing group, the NPWT group was associated with a significantly higher percentage of mean graft take at the 14th postoperative day (98.2% in the NPWT group vs. 81.2% in the conventional group; *p* < 0.05).

**Figure 1:**
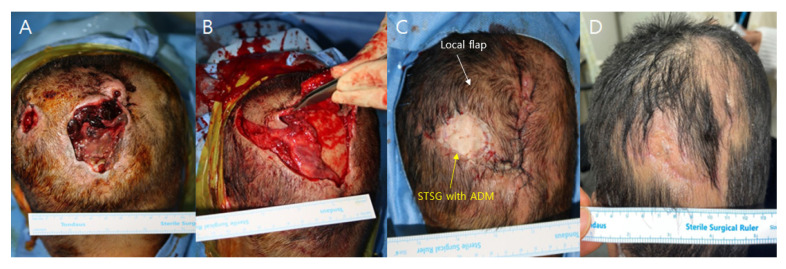
(**A**) A 52-year-old female presented with two separate occipital scalp pressure sores. (**B**) Local flap elevation, leaving deep tissue containing periosteum. (**C**) Postoperative photographic finding immediate after operation. (**D**) Postoperative photographic findings in 3-month after operation.

**Figure 2:**
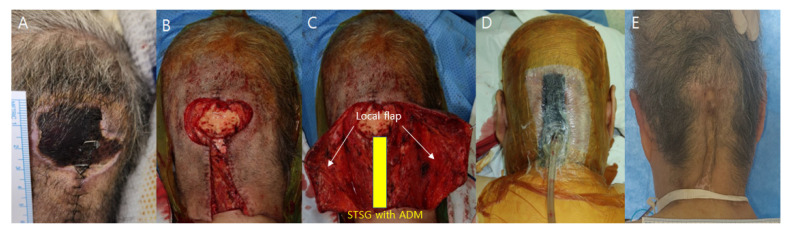
(**A**) A 75-year-old female presented with an occipital scalp pressure sore. (**B**) Bone exposure after debridement. (**C**) Local flap elevation, through subgaleal dissection. (**D**) Postoperative photographic finding with NPWT dressing. (**E**) Postoperative photographic findings 3 months after operation.

No postoperative complications such as hematoma, seroma, or wound dehiscence were identified (Figure 1 and Figure 2). However, one patient in the conventional dressing group sustained a large graft loss, which was successfully managed with 2 weeks of serial debridement and a secondary split-thickness skin graft.

- **Case Reports**
  1. Patient 1

A 52-year-old female patient presented with two separate occipital scalp pressure sores, each accompanied by full-layer skin and soft tissue necrosis, graded as IV. The patient was bedridden due to a worsening general condition following a stroke one year before. Other medical parameters, such as cardiopulmonary function and kidney and liver panels, were within the normal range. Debridement of the necrotic tissue was performed, revealing two distinct skin defects. The larger defect measured 4 × 6 cm, while the smaller one measured 2 × 2 cm. In the larger defect, there was bone exposure with a periosteum defect measuring 3 × 5 cm. For reconstruction, a scalp galeocutaneous local flap was elevated through a subgaleal dissection, leaving the deep tissue containing periosteum in its original position. The local cephalic-based galeocutaneous flap was then repositioned over the exposed bone area, and an artificial dermal matrix and split-thickness skin graft were applied over the remaining deep tissue. The procedure was performed under general anesthesia and took 45 min. NPWT was performed over both the local flap wound margin and skin graft, and the device was set to 125 mmHg on continuous mode. The NPWT dressing was changed on the 4th postoperative day and discontinued on the 7th day. The local flap showed no signs of congestion or wound problems, and the skin graft successfully took. No complications occurred during the 3-month follow-up period, and the follow-up was completed.

 2. Patient 2

A 75-year-old female patient presented with an occipital scalp pressure sore accompanied by full-thickness skin and soft tissue necrosis, graded as IV. The patient was bedridden due to neurologic sequelae following brain surgery one month ago. The patient had a previous wound problem from the surgery, which had been chronically unhealing, inflamed, and macerated. The patient had a tracheostomy and was using a mechanical ventilator with a T-cannula. Other medical parameters, including a blood laboratory panel, were within the normal range. The size of the necrotic eschar was 3.5 × 4.5 cm. Debridement was performed on the necrotic tissue and the chronic unhealing wound margin, exposing the skull bone. The skin defect measured 4 × 6 cm, with a bone exposure size of 3 × 3 cm. For reconstruction, a local bilateral occipital artery perforator-based galeocutaneous flap was elevated based on subgaleal dissection and repositioned to cover the exposed bone, while the exposed periosteum at the donor site was covered with an artificial dermal matrix and a split-thickness skin graft. The previous wound debridement site was reconstructed with the local flap. The procedure was performed under general anesthesia and took 73 min. NPWT was performed over both the local flap and skin graft, and the device was set to 125 mmHg on continuous mode. The NPWT dressing was changed on the fourth postoperative day and discontinued on the seventh day. The local flap survived with no wound problems, and the skin graft was well-taken. No complications occurred during the 3-month follow-up period, and the follow-up was completed.

## 4. Discussion

Scalp reconstruction is challenging due to the limited elasticity and spherical shape of the scalp [6]. Complicating this challenge is the occurrence of pressure sores on the occipital scalp in bedridden patients. The recurrence of pressure sores is common and requires special attention in their treatment [7]. Bedridden patients are usually restricted to a supine position and are hard to reposition. The occipital scalp is particularly vulnerable due to its dependent position [8]. First of all, medical staff and caregivers must know that avoiding pressure can prevent the occurrence or recurrence of pressure sores. Prevention is the best approach. If pressure sores, combined with tissue necrosis that cannot be healed by conservative management, occur, surgical treatment is needed. It is also important to control pressure on the postoperative wound after any type of surgery.

When reconstructing the scalp by surgery, only small defects of scalp can be closed primarily, even after wide undermining of the adjacent scalp [9]. Local flap reconstruction unavoidably requires additional incisions in a broad area, and the restricted elasticity and spherical shape of the scalp limit the feasibility of transposition techniques [10]. Tissue grafting may be considered as an option, but concerns regarding donor site morbidity and the condition of the recipient bed need to be addressed [11]. In cases of large or full layer defects, a free flap transfer may be a suitable alternative; however, there are few proper recipient vessels aside from the temporal artery and vein for scalp reconstruction with a free flap [12,13]. The occipital artery and vein are not suitable due to their extremely small size and the challenges associated with obtaining an adequate length for transplantation.

Additionally, patients with pressure sores may have poor general conditions, such as hemodynamic instability and respiratory dysfunction, making them less tolerant to prolonged surgeries involving donor site morbidities under general anesthesia [14]. In such cases, medical staff must prioritize shortening the operation and anesthesia time to minimize potential risks. Furthermore, meticulous postoperative care is crucial for flap survival after free flap surgery, including pedicle stabilization by avoiding excessive pressure or kinking [15]. However, bedridden patients and their caregivers may face challenges adhering to it due to the dependent position of the occipital scalp. For the same reason, postoperative flap monitoring is also a problem.

Split thickness skin grafts can serve as a viable option for reconstruction on any part of the body, offering advantages such as avoiding the need for additional incisions and minimizing donor site morbidity [16]. Furthermore, patients often find skin grafting more acceptable due to its shorter operation time and reduced medical burden compared with free flap reconstruction. However, in the case of bone exposure defects, the direct application of split thickness skin grafts on the bone is contraindicated due to inadequate vascular supply and a high probability of graft failure [17]. Various surgical techniques have been developed to address the reconstruction of such defects, one of which is the crane principle [18,19]. This approach involves the temporary use of a full-thickness scalp local flap positioned over the defect, followed by the placement of a cutaneous flap that is later repositioned to its original location, with deep tissue left behind. Cutaneous flap retrieval is usually done within two weeks. The remaining deep tissue serves as a well-vascularized bed for the subsequent application of a split-thickness skin graft. The transferred flap stimulates the formation of granulation tissue at the initial site of injury, creating a healthy bed for the application of the graft when the flap is eventually returned to its original position. Several studies on scalp reconstruction using the crane principle have shown favorable results [20]. The crane principle is relatively simple and can reduce financial and medical burdens. Other methods include calvaria burring and the utilization of a dermal regeneration template, such as Integra (Integra LifeSciences Corporation, Plainsboro, NJ, USA), followed by the application of a skin graft after neodermis formation [21]. Although successful outcomes have been reported with these methods, it is important to note that the total treatment time is often prolonged, requiring multiple operations. Additionally, bone burring during calvaria burring can potentially lead to complications such as increased bleeding and tissue damage.

Our proposed method builds on the crane principle by dividing the flap at the first stage, which can shorten the total treatment period and produce favorable results. Additionally, the local cutaneous flap is usually prone to shrinking due to the tension of the skin. Our method elevates the galeocutaneous flap, which includes the galea in the local flap. The inelastic nature of the galea prevents flap shrinkage. Furthermore, our method anchors the transpositioned galeocutaneous flap to the remaining deep periosteal tissue, providing appropriate tension to prevent flap shrinkage.

In our study, considering the surgical method using a local flap combined with a skin graft using NPWT, the take rate of the skin graft was higher compared with conventional dressings, without affecting the local flap status. NPWT is highly effective at promoting many types of wound healing, with VAC therapy being developed in 1997 as a specific device for NPWT [22]. NPWT reduces seroma and hematoma, decreases bacterial loading, and shortens healing time [23]. It has been applied to many types of open wounds and is also used in closed incisional negative pressure therapy (ciNPT) to reduce incision line tension and the risk of infection, decrease edema, and promote new tissue growth [24]. In particular, ciNPT has benefit in high-risk patients and incisions. The rate of surgical site infection was decreased by 50% in the ciNPT group compared with the control group, according to a recent meta-analysis. NPWT over local transpositional flaps reduces skin tension, prevents seroma and hematoma under the flap, and enhances tissue perfusion without pedicle compression [25]. Although the same flap survival rate (100%) was observed in the NPWT and conventional dressing groups in our study, NPWT resolves venous congestion of flap surgery by promoting local circulation and facilitating revascularization through neoangiogenesis, which is especially beneficial given that venous congestion is a common cause of flap failure [26,27]. In addition, an advantage of postoperative flap monitoring using NPWT is the prevention of infection-associated complications [28]. A few studies have reported adverse effects of NPWT. One study reported an adverse event that stopped NPWT prematurely because of skin blister development at the skin-dressing interface in 63% of the ciNPT group [29]. However, in our study, skin blisters did not occur in the NPWT group. Other statistically significant adverse events of NPWT were not observed.

Split thickness skin graft is vulnerable to failure owing to several factors, such as hematoma or seroma under the graft, improper fixation of the skin graft, and bacterial infection. There are various methods for covering and stabilizing skin grafts in their management. These range from traditional dressings to more innovative approaches such as non-adherent dressings with cotton pads, tie-over bolster dressings, petroleum gauze, splints, hydrocolloid and polyurethane films, fibrin glue, staplers and plastic syringes, rubber bands, and more [30]. However, a universally accepted treatment that consistently results in the successful integration of skin grafts to the recipient site has yet to be established. NPWT can immobilize and fixate the graft to the recipient bed, resulting in decreased graft loss [30]. The level of negative pressure applied is crucial to its effectiveness and must be adjusted according to the specific condition being treated. Previous meta-analyses have shown that 80 mmHg of negative pressure improves the graft take percentage [31]. In our study, NPWT was performed with 125 mmHg of negative pressure, which resulted in statistically significant and favorable results.

Furthermore, NPWT has distinct advantages in terms of patient care, particularly for bedridden patients. These patients are especially vulnerable to infections, so controlling discharge is crucial. NPWT absorbs all discharge and separates the wound from the external environment, creating a clean environment. Additionally, dressing changes can be performed less frequently compared with the conventional method, reducing the burden on patients and caregivers [32]. Frequent dressing changes can also be a source of infection. This clean environment also improves the wound healing rate and skin graft taking rate. It is also important to have proper pressure under which the skin graft can be immobilized without causing additional skin injury. With conventional dressing, improper pressure distribution can cause skin injuries or other pressure sores. NPWT adequately controls pressure and minimizes the risk of pressure ulcers. Our surgical method is a good indication for applying NPWT. Local flap wound healing and split thickness skin graft taking are promoted by NPWT. Furthermore, pressure sore patients are usually at high risk of surgical site infection and developing another pressure sore [33]. NPWT can be a good tool for preventing surgical site infection or pressure sores [28]. In addition, the reason we prefer skin graft instead of using multiple local flaps is that in most patients with unconscious or position limitation, reducing the operative area can prevent complications such as flap failure. However, in areas where the defect size is too large or tissue laxity is limited, reconstruction was inevitably performed using multiple flaps and the skin graft technique.

The flexibility and overall condition of the skin is largely determined by the dermis layer [34]. Dermal substitutes are an excellent option for replacing a patient’s damaged dermis. The process of resurfacing a dermal defect can be enhanced by using dermal regeneration templates, which increase the flexibility of the grafted area and improve the overall quality of the skin [35]. The most popular dermal substitute on the market is Integra. However, Integra takes time to generate a neodermis—approximately two weeks or more. Our methods are designed to shorten the operation time and total treatment period. This method of processing and using an acellular dermal matrix such as Alloderm is an excellent alternative to complex dermal products such as Integra, eliminating the need for a two-stage operation. Our study used a one-stage type of acellular dermal matrix and achieved favorable outcomes. Using an acellular dermal matrix derived from allografts in combination with thin split-thickness skin autografts in a single stage is an effective, cost-effective, and favorable option for covering deep wounds or when thick skin grafts are needed. According to senior operator’s clinical experience, single split-thickness skin graft does not guarantee sufficient durability for patients with unclear consciousness or mobility difficulties. Consequently, the acellular dermal matrix was used in all patients in this study and the postoperative results were relatively positive. The effect of NPWT on dermal substitutes has been reported in previous studies. The usage of Integra with NPWT showed favorable results according to previous reports [36]. Recently, split-thickness skin grafts above acellular dermal matrices with NPWT also showed favorable results, similar to the results of this study [37,38,39].

This study has a few limitations. Firstly, although the study was conducted by single senior surgeon, the number of patients was small. Secondly, it is possible that this it affected the surgical outcomes such as the graft take rate because the variables for the underlying diseases or compliance of patients were not controlled. Furthermore, the defect size of the patients included in this study was less than 24cm^2^. The authors propose free tissue transfer for larger defects with bone exposure or split thickness skin graft for defects without bone exposure. Lastly, costs for the dressing material were not assessed in this study. According to previous reports considering the cost-effectiveness of NPWT for postoperative management, this can be said to be a limitation of this study.

## 5. Conclusions

As the aging population increases, occipital pressure sores are drawing increasing attention as an important disease. A novel surgical method with NPWT enabled an efficient and safe treatment option for patients with occipital pressure sores. This novel surgical technique and postoperative management is a promising candidate for future recognition as the gold standard for pressure sore treatment.

## Figures and Tables

**Table 1:** Comparison of results among both treatment methods.

	NPWT Group	Conventional Group	*p* Value
Number of patients	24	22	
Age, years, mean ± SD	72.4 ± 7.1	73.8 ± 6.6	0.686
Defect size, cm^2^, mean ± SD	16.2 ± 1.6	18.4 ± 2.1	0.372
Follow up, months, mean ± SD	6.1 ± 0.1	5.9 ± 0.1	0.891
Flap surface, cm^2^, mean ± SD	60.5 ± 5.3	57.2 ± 5.1	0.709
Operation time, min, mean ± SD	85.7 ± 9.7	80.4 ± 8.4	0.841
Flap survival rate (%)	100	100	0.5
Graft taken, %, mean ± SD	98.2 ± 2.8	81.2 ± 3.6	0.014
Other Complications *	0	0	0.5

* Other complications: hematoma, seroma, and wound dehiscence.

## Data Availability

Not applicable.
